# Cognitive Aging: New Insights Into How Our Brains Age

**DOI:** 10.1093/geroni/igaa057.2626

**Published:** 2020-12-16

**Authors:** Coleen Murphy, Saul Villeda

## Abstract

Maintaining quality of life with age is as important as slowing human aging. Slowing cognitive decline will be a critical aspect of keeping older adults healthy as we extend lifespan. To that end, identifying the molecular regulators of neuronal health, including learning and memory, in model systems that can later be applied to humans is an important step. In this symposium, we will hear about work on cognitive decline and its prevention being done in a variety of model systems.

